# Supplementary material for: The long-term effect of short point of care ultrasound course on physicians’ daily practice
Source: PLoS One. 2020 Nov 20;15(11):e0242084. doi: 10.1371/journal.pone.0242084 (PMC7678973; doi:10.1371/journal.pone.0242084)
Supplement: S1 Appendix — (DOCX) [file pone.0242084.s002.docx]

**S1 Appendix.**

**Point of Care Ultrasound Curriculum**

**Learning Objectives:**

**Upon completion of this course, participants will be able to:**

- Perform focused echocardiographic exams
- Differentiate between normal and abnormal echocardiographic exams
- Screen for and identify 6 major causes of shock: hypovolemic, septic, temponade, pulmonary embolism, cardiogenic and pneumothorax
- Perform chest ultrasound for the diagnosis of pneumothorax, pulmonary edema and pneumonia
- Perform the FAST exam for diagnosis of intra-abdominal bleeding in the setting of blunt trauma
- Understand challenges involved in incorporating focused cardiac ultrasound during cardio-pulmonary resuscitation efforts.

**Program Schedule:**

- Basic Knobology
- Ultrasound Anatomy of the Heart: Basic TTE views
- Principles of Doppler Ultrasound
- Assessment of Left Ventricular Systolic Function
- Assessment of Right Ventricular Systolic Function
- Assessment of Tamponade Physiology
- Assessment of Pulmonary Embolism
- Chest Ultrasound: Pneumothorax, Pleural Effusion and consolidations
- Overall approach to shock and the Focused Echocardiographic Evaluation in Life Support (FEEL) algorithm
- Basic Assessment of Valvular Dysfunction
- Ultrasound Guided Vascular Access
- Evaluation of Chest Trauma
- Pitfalls and Limitations of Focused Echocardiography
- Clinical Cases, adjourn and certificates
- Hands- on Training (4 sessions)
- Simulator Training: Applying FEEL in resuscitation
- FAST exam
