# Supplementary material for: The long-term effect of short point of care ultrasound course on physicians’ daily practice
Source: PLoS One. 2020 Nov 20;15(11):e0242084. doi: 10.1371/journal.pone.0242084 (PMC7678973; doi:10.1371/journal.pone.0242084)
Supplement: S2 Appendix — (DOCX) [file pone.0242084.s003.docx]

**S2 Appendix.**

'Point of Care Ultrasound' course- Questionnaire

1. What is your frequency of US utilization post- POCUS course?

Not using/  Little use / Reasonable use/  Multiple use

1. To what extent do you believe incorporating POCUS into your practice will improve the care you provide to patients?
    Strongly disagree/ Disagree/ Neutral/ Agree/ Strongly agree
2. To what extent do you agree with the following statement: A short Point of Care ultrasound course improve diagnostic skills?

Strongly disagree/ Disagree/ Neutral/ Agree/ Strongly agree

1. To what extent do you agree with the following statement: Point of care ultrasound is a diagnostic modality that can be used by clinicians to provide more accurate and quicker diagnosis for several disease processes?

Strongly disagree/ Disagree/ Neutral/ Agree/ Strongly agree

1. Do you think incorporating POCUS as a part of your daily practice may influence on your patients' outcome and recovery? (e.g. length of hospital stays, admission rate etc.)?

Strongly disagree/ Disagree/ Neutral/ Agree/ Strongly agree

# Do you think POCUS course should be an integral part of both residents’ and specialists’ medical training?

Strongly disagree/ Disagree/ Neutral/ Agree/ Strongly agree

1. Do you agree with the following statement: Short POCUS course may shorten time to diagnosis thus may reduce morbidity?
     Strongly disagree/ Disagree/ Neutral/ Agree/ Strongly agree
2. Have your ultrasound skills improved due to this course?

Strongly disagree/ Disagree/ Neutral/ Agree/ Strongly agree

1. How comfortable do you feel with your understanding of the ultrasound machine and your ability to operate it within different clinical scenarios after the course?
   Not at all/ Minimally/ Moderately/  Greatly /  Extremely
2. How comfortable do you feel with your understanding of the capabilities and limitations of POCUS?
    Strongly disagree/ Disagree/ Neutral/ Agree/ Strongly agree
3. How likely are you to integrate POCUS into your routine clinical care after the course?
   Not at all/ Minimally/ Moderately/  Greatly /  Extremely
4. Have you ever had similar training in this field??

Yes / No

1. Would you recommend this course to your colleagues?
    Yes / No
