# Supplementary material for: The long-term effect of short point of care ultrasound course on physicians’ daily practice
Source: PLoS One. 2020 Nov 20;15(11):e0242084. doi: 10.1371/journal.pone.0242084 (PMC7678973; doi:10.1371/journal.pone.0242084)
Supplement: S3 Appendix — (DOCX) [file pone.0242084.s004.docx]

**S3 Appendix. Comparison of participants’ answers stratified by year of the US course**

| **Questions** |  | **2014-2015** | **2016-2017** | **P value** |
| --- | --- | --- | --- | --- |
| **Question 2** | 0-2 | 6 (23.1%) | 22 (24.4%) | 0.88 |
|  | 3-4 | 20 (76.9%) | 68 (75.6%) |  |
| **Question 3** | 0-2 | 4 (15.4%) | 14 (15.6%) | 0.98 |
|  | 3-4 | 22 (84.6%) | 76 (84.4%) |  |
| **Question 4** | 0-2 | 4 (15.4%) | 5 (5.6%) | 0.09 |
|  | 3-4 | 22 (84.6%) | 85 (94.4%) |  |
| **Question 5** | 0-2 | 19 (73.1%) | 60 (66.7%) | 0.53 |
|  | 3-4 | 7 (26.9%) | 30 (33.3%) |  |
| **Question 6** | 0-2 | 2 (7.7%) | 4 (4.4%) | 0.50 |
|  | 3-4 | 24 (92.3%) | 86 (95.6%) |  |
| **Question 7** | 0-2 | 7 (26.9%) | 20 (22.2%) | 0.61 |
|  | 3-4 | 19 (73.1%) | 70 (77.8%) |  |
| **Question 8** | 0-2 | 7 (26.9%) | 10 (11.1%) | 0.04 |
|  | 3-4 | 19 (73.1%) | 80 (88.9%) |  |
| **Question 9** | 0-2 | 6 (23.1%) | 19 (21.1%) | 0.83 |
|  | 3-4 | 20 (76.9%) | 71 (78.9%) |  |
| **Question 10** | 0-2 | 5 (19.2%) | 11 (12.2%) | 0.36 |
|  | 3-4 | 21 (80.8%) | 79 (87.8%) |  |
| **Question 11** | 0-2 | 15 (57.7%) | 35 (38.9%) | 0.08 |
|  | 3-4 | 11 (42.3%) | 55 (61.1%) |  |
| **Question 12** | No | 23 (88.5%) | 69 (76.7%) | 0.19 |
|  | Yes | 3 (11.5%) | 21 (23.3%) |  |
| **Question 13** | No | 5 (19.2%) | 10 (11.1%) | 0.27 |
|  | Yes | 21 (80.8%) | 80 (88.9%) |  |
